# Lead Screening for HIV-1 Integrase (IN) Inhibited by Traditional Chinese Medicine

**DOI:** 10.1155/2014/479367

**Published:** 2014-06-11

**Authors:** Tzu-Chieh Hung, Wen-Yuan Lee, Kuen-Bao Chen, Yueh-Chiu Chan, Calvin Yu-Chian Chen

**Affiliations:** ^1^Department of Biomedical Informatics, Asia University, Taichung 41354, Taiwan; ^2^School of Medicine, College of Medicine, China Medical University, Taichung 40402, Taiwan; ^3^Department of Neurosurgery, China Medical University Hospital, No. 2 Yude Road, North District, Taichung 40447, Taiwan; ^4^Department of Anesthesiology, China Medical University Hospital, Taichung 40447, Taiwan; ^5^Research Center for Chinese Medicine and Acupuncture, China Medical University, Taichung 40402, Taiwan

## Abstract

Human immunodeficiency virus causes the acquired immunodeficiency syndrome (AIDS) and becomes a serious world-wide problem because of this disease's rapid propagation and incurability. Integrase strand transfer inhibitors (INSTIs) supports HIV have rapid drug resistance for antitreatment. Screening the traditional Chinese medicine (TCM) database by simulating molecular docking and molecular dynamics may select molecular compounds to inhibit INSTIs against HIV drug resistance. (S)-cathinone and (1S,2S)-norpseudoephedrine are selected based on structure and ligand-based drugs are designed and then get higher bioactivity predicted score from SVM than Raltegravir and other TCM compounds. The molecular dynamics are helpful in the analysis and detection of protein-ligand interactions. According to the docking poses, hydrophobic interactions and hydrogen bond variations define the main regions of important amino acids in integrase. In addition to the detection of TCM compound efficacy, we suggest (1S,2S)-norpseudoephedrine is better than the others based on the analysis of interaction and the effect on the structural variation.

## 1. Introduction

The acquired immunodeficiency syndrome (AIDS) is caused by a retrovirus, the Human immunodeficiency virus (HIV) [1–4]. In AIDS, the immune system is inhibited by the virus, which makes patients have more opportunities for deadly infections and cancers. The HIV virus is transmitted via unprotected sexual intercourse [5, 6], contaminated medical equipment [7, 8], bodily fluids, and vertical infection (pregnancy, delivery, or breastfeeding) [9, 10].

AIDS has caused nearly thirty-six million deaths since the first case in 1981 and there were approximately seventy-five million carriers as recorded by UNAIDS (http://www.unaids.org/en/resources/campaigns/globalreport2013/factsheet/). There are still no vaccines or drugs available to kill all the viruses in body; thus, highly active antiretroviral therapy (HAART) had identified the standard of care for patients with advanced infection in these years [11] which decreases the patient's total burden of HIV success by the complex transcription inhibitors but this treatment is expensive.

HIV-1 integrase (IN) is an essential enzyme which catalyzes the integration of the viral DNA into the host cell genome. According to human without the enzyme integrase, the inhibitor of HIV-1 integrase becomes a promising therapeutic target for AIDS. After the rapid drug resistance of HIV-1 integrase had been found, several drugs approved by the FDA lost their efficacy. There is a reference that indicates the drug target site of integrase and explores the molecular mechanism of drug resistance [12]. Based on this research, the drug inhibiting integrase and preventing the resistance is feasible.

Computer-aided drug design (CADD) is an* in silico* simulation technique to screen for molecular compounds by the structure and to predict the biological activity of drug character. In comparison with traditional drug design, CADD has the advantages of both greater speed and lower cost. The structure-based drug design and ligand-based drug design are two major application areas of CADD. We used CADD to investigate the molecular simulation in drug design on the basics of structure-based drug design and molecular dynamics [13–18].

In these two decades, the personalized medicine and biomedicine are important knowledge [19, 20] for the mutation [21, 22], the pathway [23, 24], the cause for special disease [25–27], and the clinical diagnosis [28]. The traditional Chinese medicine (TCM) is identified as one of personalized medicines. TCM has an important role in Asia, especially in Chinese culture. The TCM Database@Taiwan (http://tcm.cmu.edu.tw/) [29] is the largest traditional Chinese medicine database established in 2011. There are 2D chemical structures, 3D chemical structures, bioactivity, and molecular information for over 61,000 compounds of traditional Chinese medicinal herbs in this database. Since 2011, the TCM Database@Taiwan has made successful discoveries of novel compounds for cancer treatment [30–33], stroke prevention [34], EGFR inhibition [35], inflammation inhibition [36], pain relief [14], and antivirals [37–41]. The TCM Database@Taiwan could be valuable for TCM application and drug design with the application of the website [42] and the cloud computing platform [43].

In this research, we screen a possible compound against HIV from the TCM Database@Taiwan. We use the molecular docking screening to select ligands, and then we apply molecular dynamics (MD) simulation to investigate variations from protein ligand interactions. This program may contribute to the evaluation of the effect of integrase inhibition.

## 2. Materials and Methods

### 2.1. Data Set

Accelrys Discovery Studio 2.5 (DS 2.5) was used as a docking platform for the molecular simulations. A total of 61,000 TCM compounds had been downloaded from the TCM database (http://tcm.cmu.edu.tw/). The HIV-1 integrase crystal structure was obtained from Protein Data Bank (PDB ID: 2B4J), and Raltegravir, as control drug, helped design the docking site [12].

### 2.2. Disorder Protein Detection

We take the sequence of protein structure from Uniprot (http://www.uniprot.org/) and docking site to predict the disorder region by the Database of Protein Disorder (DisProt: http://www.disprot.org/) [44]. The result of prediction could analyze the character of the docking site and the efficacy of the drug.

To compare the region of the disorder protein and the docking sites, we could assess the protein-ligand interaction and drug efficacy effect from disorder.

### 2.3. Molecular Docking

The docking simulation used the LigandFit [45], a receptor-rigid docking algorithm program in Discovery Studio 2.5 (DS 2.5), module in the force field of CHARMM [46] to dock Raltegravir and TCM compounds to HIV integrase. The docking site of HIV integrase was identified by the research [12, 47].

### 2.4. Ligand-Based Prediction

Bioactivity prediction was assessed by the MLR and SVM models. The pIC_50_ of model drugs for integrase was set as the template to assist with model assessment [48]. Before creating the prediction model, the descriptors of these ligands were evaluated by the genetic approximation (GA) algorithm of the Calculate Molecular Properties module in Accelrys Discovery Studio 2.5 [49].

The MLR was established by the five descriptors and the MatLab Statistics Toolbox was used to select the ligand based on activity [50]. After prediction, the result should be detected by the Leave One Out Validation [51].

Setting the SVM model used the same ligand template and the production of descriptors and descriptors should be normalized to transform the range from −1 to 1. Screening the best training model was based on the Five-fold Cross Validation [52].

The results were ranked by the score of SVM prediction. The top compounds were selected with the protein as complex to analyze the hydrophobic interactions by Ligplus [53, 54] and then be submitted to the molecular dynamics simulation.

### 2.5. Molecular Dynamics Simulation

Before applying MD simulation, selected ligands must be reprepared based on the reference force field [55] of GROMACS 4.5.5 [56] through SwissParam (http://swissparam.ch/) [57]. The HIV integrase combines with ligands going into the buffer (or solution) simulation box. This cubic box was solvated with the TIP3P water model in which sodium and chloride ion were added to neutralize complex charges within a minimum distance of 1.2 Å from the complex to box. The complex was minimized with the steepest descent method for 5,000 steps, and then the last structure with lowest energy was transferred to MD simulation. The electrostatic interactions were calculated based on the particle-mesh Ewald (PME) method [58], in which situation, each time step was 2 fs and the numbers of steps were 10,000,000 times. According to the Berendsen weak thermal coupling method, the equilibration was under the 100 ps constant temperature (PER ensemble). The total simulation time of MD was 20 ns. The Gromacs 4.5.5 had protocols to analyze MD trajectories, RMSD, and energy variations of the complex.

## 3. Results and Discussion

### 3.1. The Detection of Disorder Protein

**Figure 1:**
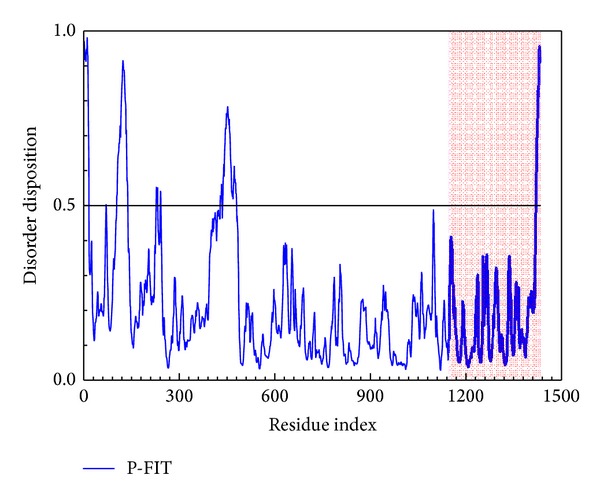
The disorder region prediction and binding site detection. The blue curve is the disorder disposition of each amino acid, and pink regions are the residues of the important amino acids.

The disorder protein is an unstructured protein which makes the drug dock to protein hardly and the complex will stabilize with difficultly. But some references [41, 42] indicate that the interaction with the disorder region might cause lower side effect than with the widespread domain; thus, the disorder region cannot be defined as a bad docking site for selection. The disorder regions of HIV integrase are defined as having a disposition of over than 0.5 (Figure 1) which indicates that the docking and functional region do not consist of disorder regions, thus the ligand docking to the selected site has a weaker effect from disorder protein.

### 3.2. Candidate Compounds Detection

**Figure 2:**
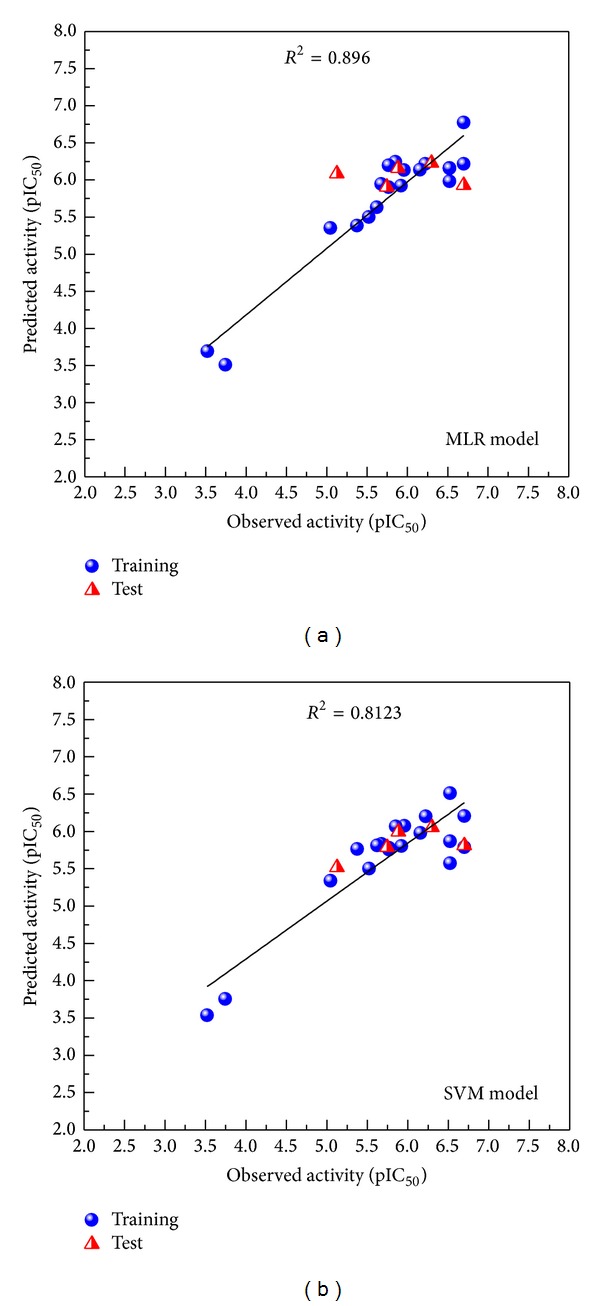
Relation of observed activity (pIC50) and predicted activity (pIC50), (a) MLR and (b) SVM.

The ligand based prediction should be detected correctly (Figure 2). The correlation coefficient (r2) of both SVM and MLR is higher than 0.8 which means our bioactivity prediction is credible and the selected compounds may have the same efficacy as the template drug with the function of integrase inhibition. The top two TCM compounds can be selected (Table 1). These TCM compounds, (S)-cathinone and (1S,2S)-norpseudoephedrine selected from TCM database, are both extracted from the herb* Ephedra sinica stapf* which was defined as anti-HIV herb [59–61]. Cathinone has reported the function for HIV [62] and immunity [63]. Thus we suggest the selected compounds might be against HIV through the inhibition of integrase.

**Figure 3:**
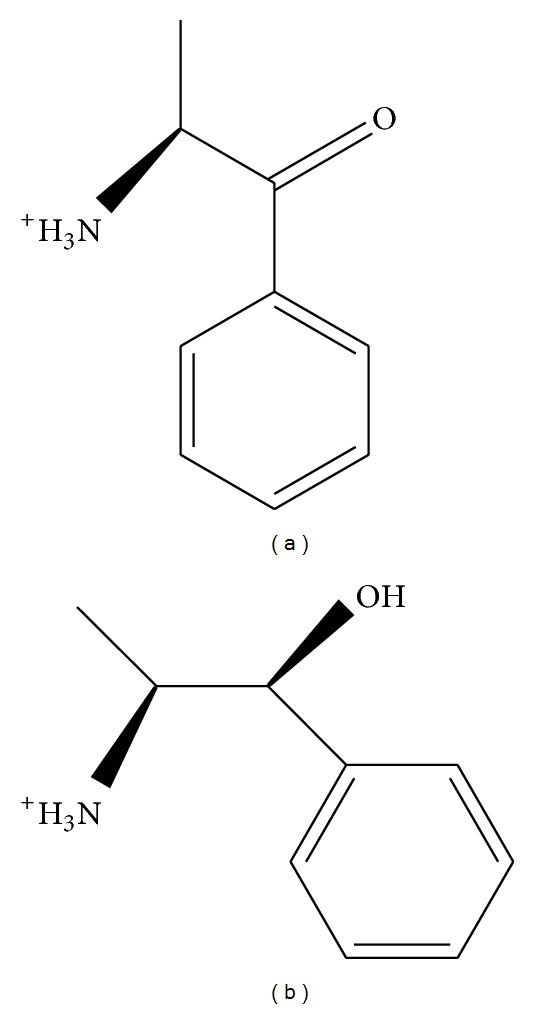
The 2D structure of the control and candidate TCM compounds, (a) (S)-cathinone and (b) (1S,2S)-norpseudoephedrine.

**Figure 4:**
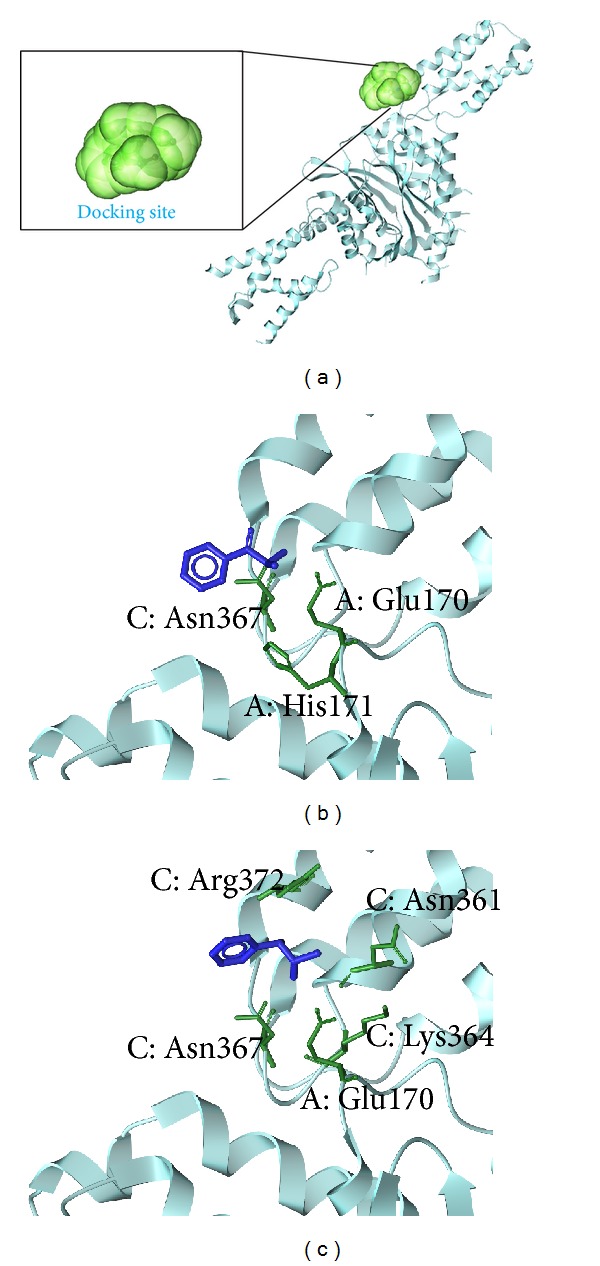
The docking poses of the ligands, (a) the crystal structure of integrase and the docking site, (b) (S)-cathinone, and (c) (1S,2S)-norpseudoephedrine.

The structure of the candidate compounds (Figure 3) and the docking poses sign, the docking site, and the amino acid neighbors by ligands is shown (Figure 4). From this result, we observe compounds interact the A and C substructures of whole HIV integrase (A, B, C, and D subunits).

**Figure 5:**
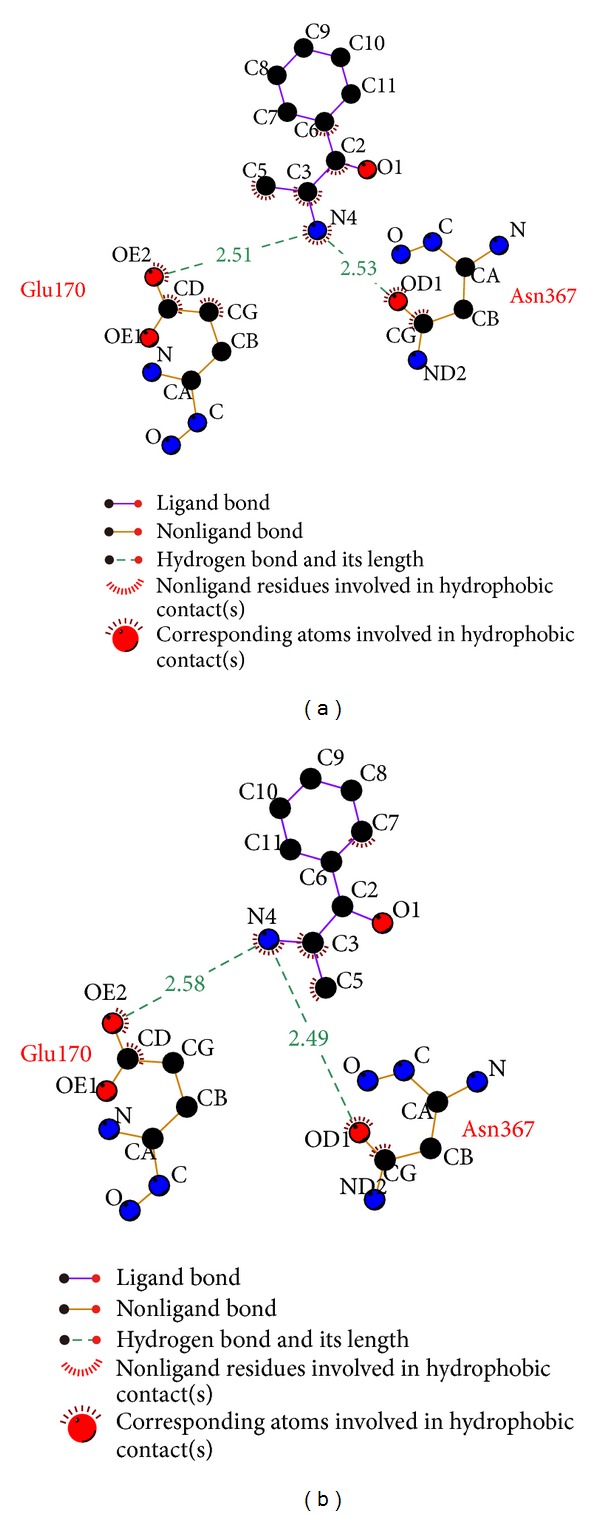
Ligplot illustrates the hydrophobic interactions, (a) (S)-cathinone and (b) (1S,2S)-norpseudoephedrine.

After the hydrophobic interaction is analyzed by Ligplus, Glu170 of A unit and Asn367 of C are also found the interaction with ligand (Figure 5). These results may present that Glu170 of A unit and Asn367 of C have an effect on HIV integrase.

### 3.3. Molecular Dynamics Simulation

**Figure 6:**
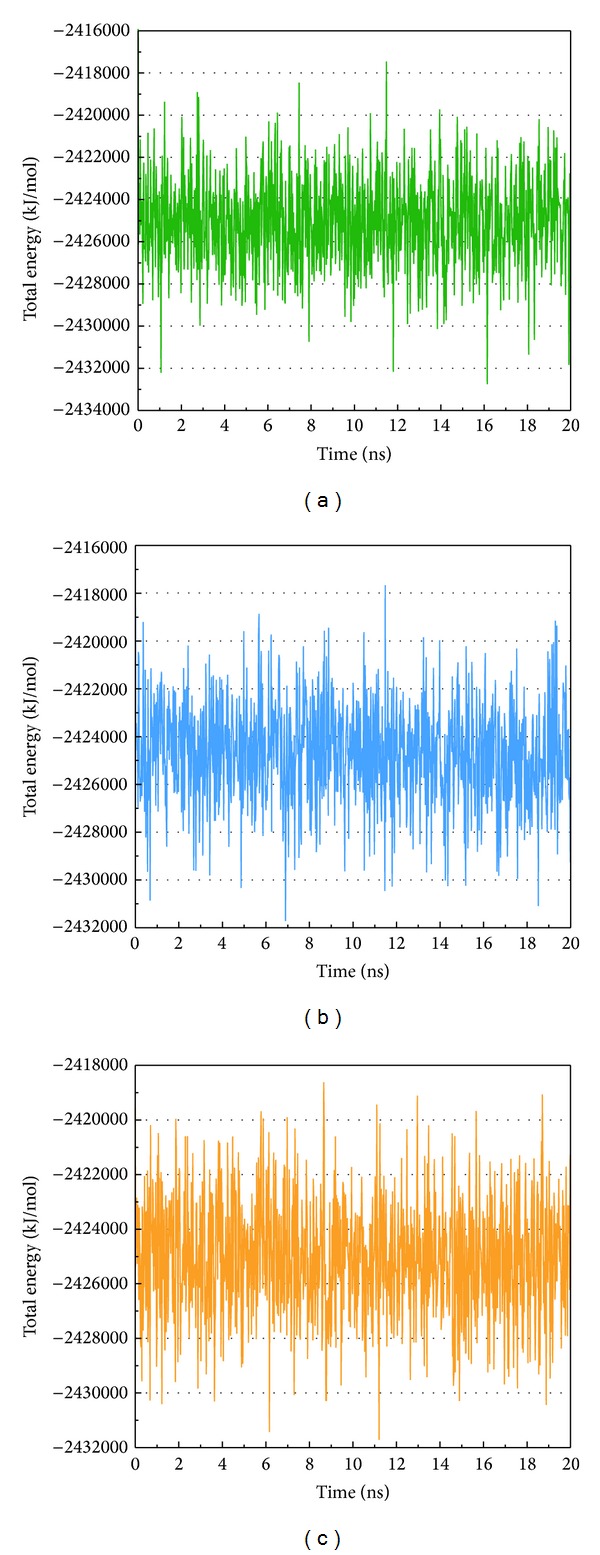
Measures of total energy variation, (a) (S)-cathinone, (b) (1S,2S)-norpseudoephedrine, and (c) apo (unbound protein).

**Figure 7:**
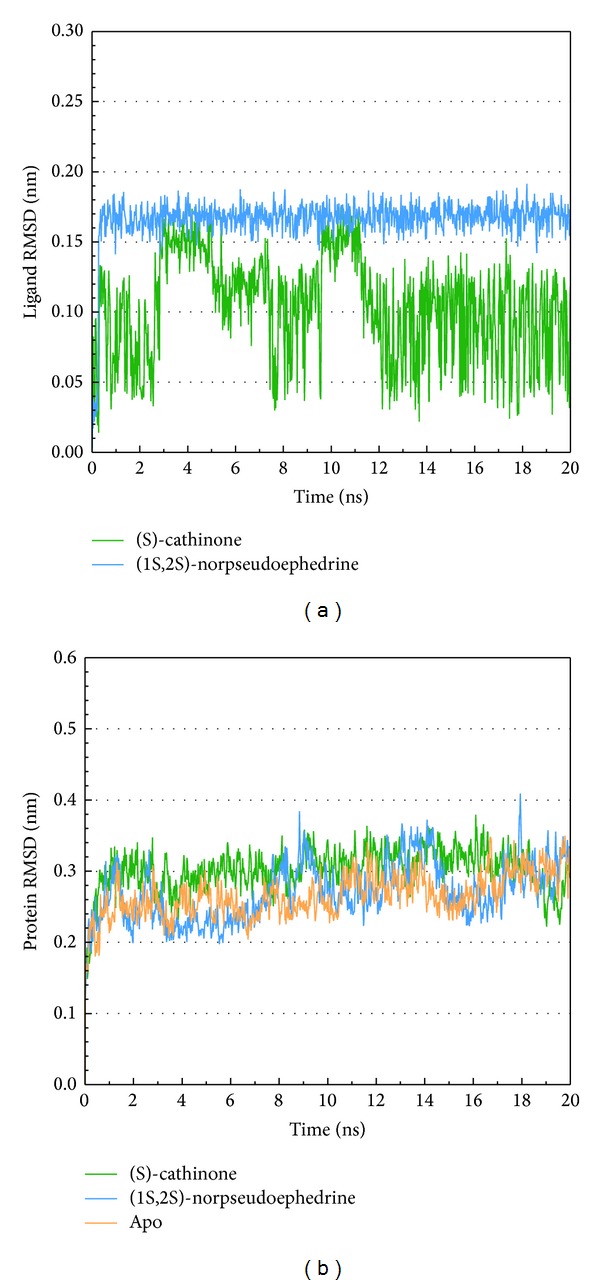
Measures of the RMSD variation; (a) is ligand RMSD and (b) is protein RMSD.

The total energy variation of complex and apo (unbound protein) range between −2428 and 2422∗10^3^ KJ/mol and have a tendency towards 2424∗10^3^ KJ/mol (Figure 6). The RMSD calculate averagement of residue position variation caused by the protein-ligand interaction (Figure 7). According to lowest energy and RMSD, we find the structure of apo is more stable than the complex before 16 ns. Thus, we suggest the integrase may be inhibited while compounds interact to make the structure unstable.

**Figure 8:**
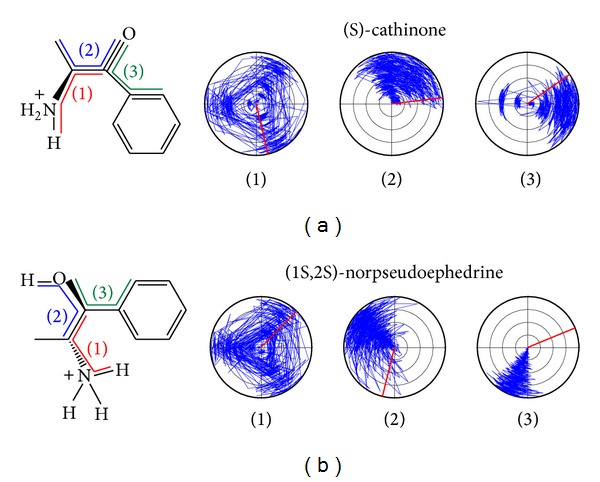
Torsion angles of compounds during MD, (a) (S)-cathinone and (b) (1S,2S)-norpseudoephedrine.

The torsion of compounds could help us understand the interaction site while the compound affects protein (Figure 8). In this result, the main structure is less variant and the side is larger which might affect the interaction of different amino acids and then the direction of torsion turn.

**Figure 9:**
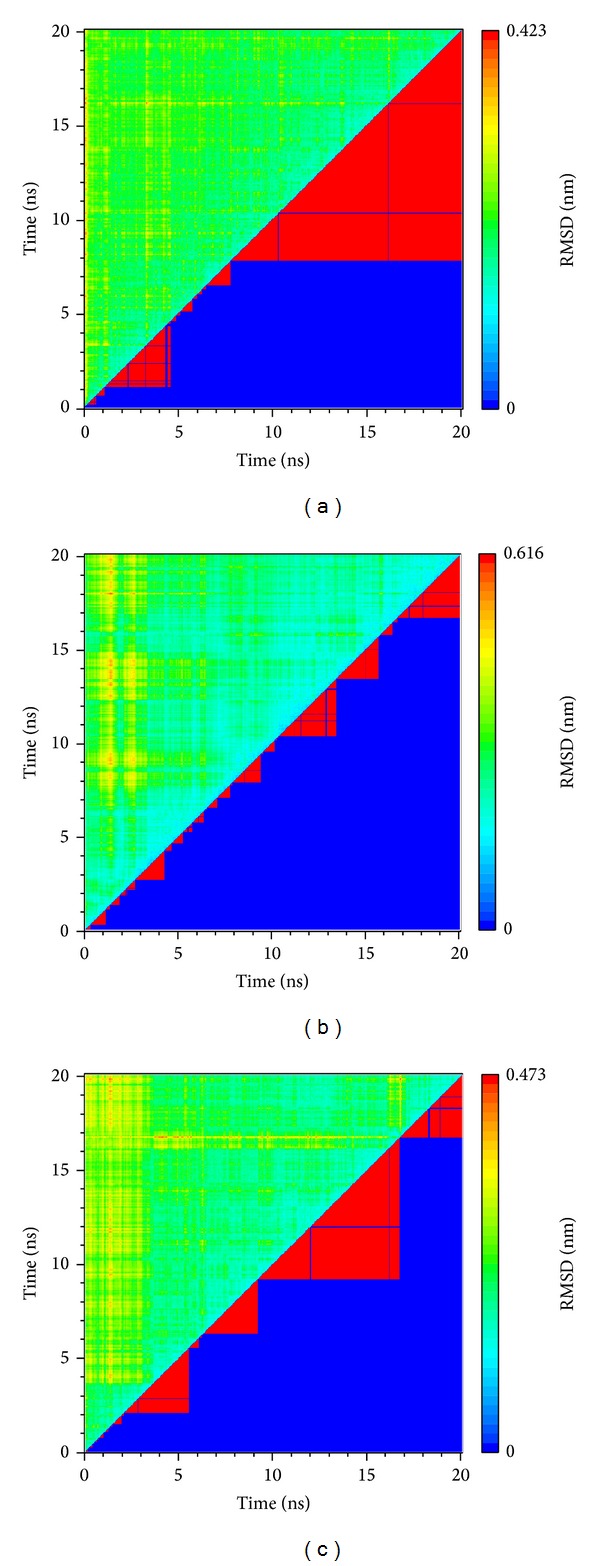
Clustering the ligand-protein interaction, (a) (S)-cathinone, (b) (1S,2S)-norpseudoephedrine, and (c) apo.

The clustering is calculating the RMSD variation and divides the similarity to the same group thus the structure may be the same in a group (Figure 9). For this reason, the largest group at the end means this complex might be more stable or the simulation might be balanced. In this result, we find (S)-cathinone is more stable than the others and the (1S,2S)-norpseudoephedrine has the largest group in this clustering. We suggest that the different kinds of two compounds' interaction may indicate two kinds of influence that the (S)-cathinone will target as complex to interact the protein, and the (1S,2S)-norpseudoephedrine might make the structure unstable to inhibit integrase.

**Figure 10:**
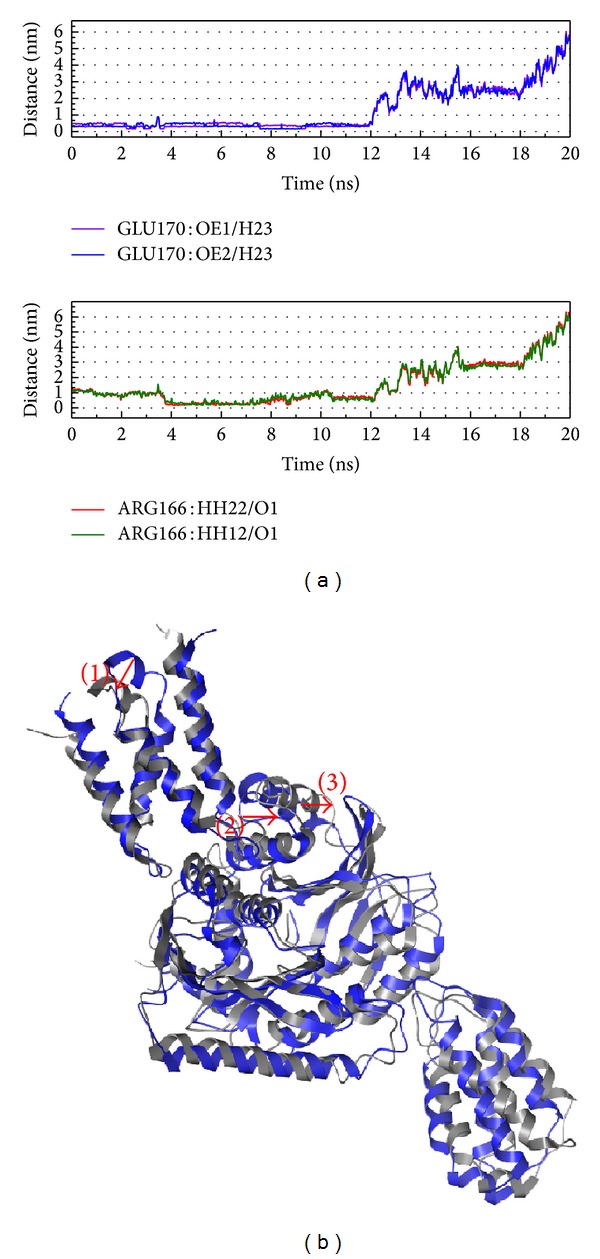
The variation of (S)-cathinone and integrase complex in MD simulation, (a) H-bond variation and (b) structure variation. The (1)–(3) red color indicates the difference through MD.

**Figure 11:**
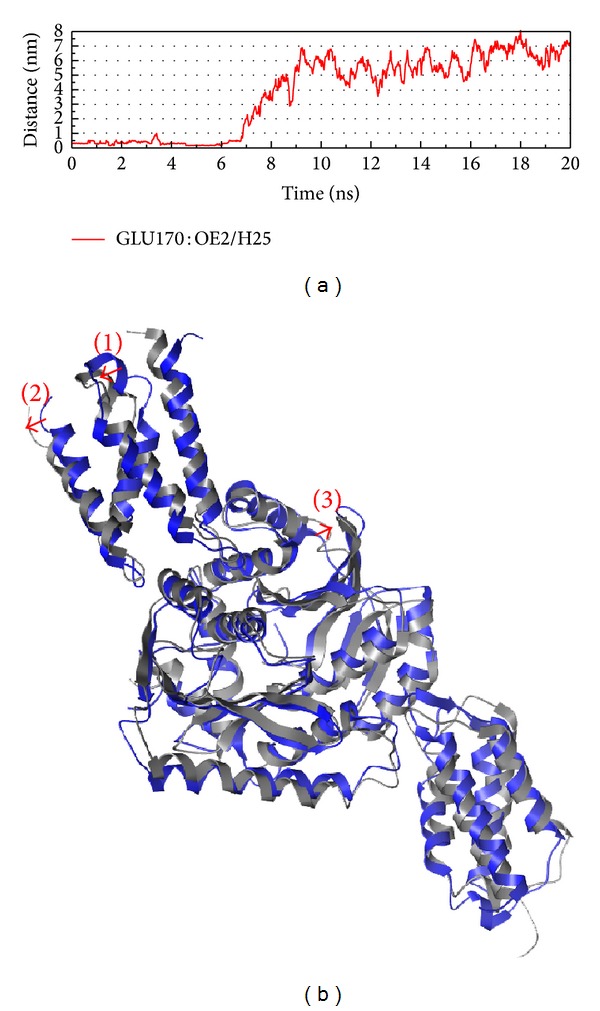
The variation of (1S,2S)-norpseudoephedrine and integrase complex in MD simulation, (a) H-bond variation and (b) structure variation. The (1)–(3) red color indicates the difference through MD.

To compare the H bond and structure variation in MD 0 ns and 20 ns could help the definition of interaction (Figures 10 and 11). In H bond variation, we find the distance is longer which means the compounds move away from the docking site. From this situation, we suggest the integrase is important for virus and this enzyme wants to prevent the function inhibited; thus, this kind of protein will make weak interaction between ligands. Even in the short time interaction, we also could find the structure variation (we only signed around docking site focus on our discussion, but the variation of other sites might have more function effect).

**Figure 12:**
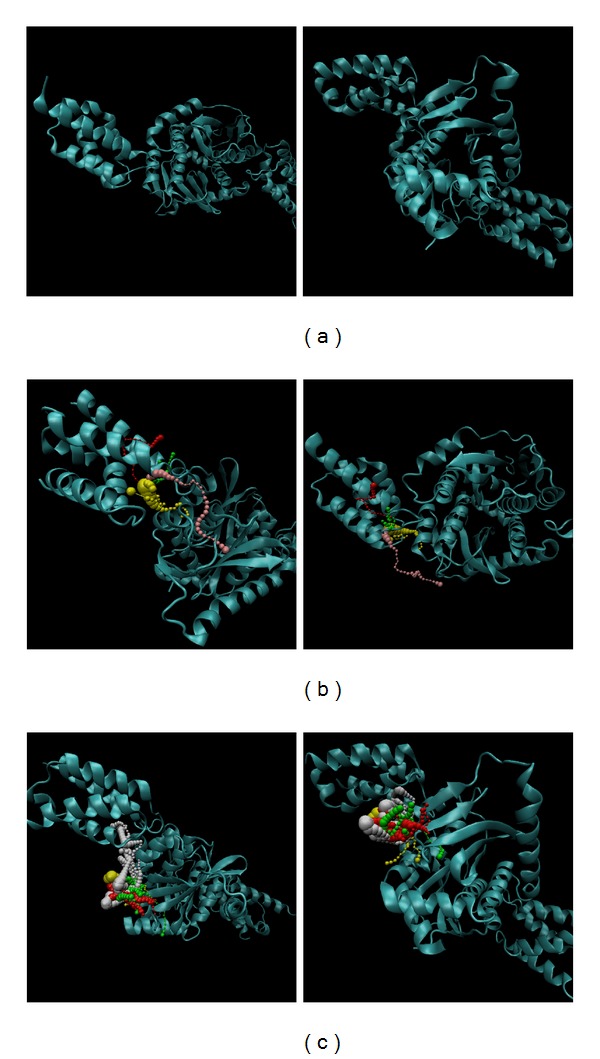
The pathway calculation of integrase complex as a path for ligand in MD simulation, (a) apo, (b) (S)-cathinone, and (c) (1S,2S)-norpseudoephedrine.

The pathway could help understanding the path for ligand interaction with protein could be defined based on the calculation of caver 3.0 to determine the interpath protein path during MD simulation (Figure 12) [64]. Most of the pathways are not inside the protein, then the present interaction is located on the surface and the ligand could not target the protein closely; thus, the efficacy will decrease.

## 4. Conclusion

Based on the above discussion, we find the top two TCM compounds, (S)-cathinone and (1S,2S)-norpseudoephedrine, can have an effect on HIV integrase against HIV infection. The ligand impacts integrase through hydrophobic interactions and H-bonds but the protein tries to prevent these influences which make the structure vary and affect the function. With these analyses of interaction and discussion the character of integrase prevents ligand targeting. We suggest both the herb* Ephedra sinica stapf* and (1S,2S)-norpseudoephedrine may have a better effect on the inhibition of integrase based on the larger variation than other compounds and the fact that the result for protein is more unstable.

## Figures and Tables

**Table 1:** The score of molecular docking and bioactivity of prediction.

Name	DockScore	SVM	MLR	Herb
(S)-cathinone	87.568	7.513	8.058	*Ephedra sinca stapf *
(1S,2S)-norpseudoephedrine	80.074	7.262	6.689	*Ephedra sinca stapf *
Octopamine	81.861	7.093	6.950	*FRUCTUS AURANTII *
Noradrenaline	95.291	6.955	5.746	*Portulaca oleracea *
P-synephrine	77.385	6.814	4.015	*FRUCTUS AURANTII *
3,4,5-Trimethoxy benzeneethanamine	83.352	6.738	13.406	*Myristica fragrans *
D77*	74.525	6.667	−11.476	
Raltegravir*	43.285	6.482	−9.812	

*Control.
